# Morphological, Physical, and Mechanical Properties of Sugar-Palm (*Arenga pinnata* (*Wurmb*) *Merr*.)-Reinforced Silicone Rubber Biocomposites

**DOI:** 10.3390/ma15124062

**Published:** 2022-06-08

**Authors:** Siti Humairah Kamarul Bahrain, Nik Rozlin Nik Masdek, Jamaluddin Mahmud, M. N. Mohammed, S. M. Sapuan, R. A. Ilyas, Abdullah Mohamed, Mohamed A. Shamseldin, Anas Abdelrahman, M. R. M. Asyraf

**Affiliations:** 1 School of Mechanical Engineering, College of Engineering, Universiti Teknologi MARA, Shah Alam 40450, Selangor, Malaysia; nikrozlin@uitm.edu.my (N.R.N.M.); jm@uitm.edu.my (J.M.); 2 Mechanical Engineering Department, College of Engineering, Gulf University, Sanad 26489, Bahrain; dr.mohammed.alshekhly@gulfuniversity.edu.bh; 3 Laboratory of Biocomposite Technology, Institute of Tropical Forestry and Forest Products (INTROP), Universiti Putra Malaysia, Serdang 43400, Selangor, Malaysia; sapuan@upm.edu.my; 4 Advanced Engineering Materials and Composites Research Centre (AEMC), Department of Mechanical and Manufacturing Engineering, Faculty of Engineering, Universiti Putra Malaysia, Serdang 43400, Selangor, Malaysia; 5 School of Chemical and Energy Engineering, Faculty of Engineering, Universiti Teknologi Malaysia, Johor Bahru 81310, Johor, Malaysia; 6 Centre for Advanced Composite Materials (CACM), Universiti Teknologi Malaysia, Johor Bahru 81310, Johor, Malaysia; 7 Research Centre, Future University in Egypt, New Cairo 11835, Egypt; mohamed.a@fue.edu.eg; 8 Department of Mechanical Engineering, Faculty of Engineering & Technology, Future University in Egypt, New Cairo 11845, Egypt; mohamed.abelbbar@fue.edu.eg; 9 Mechanical Engineering Department, Faculty of Engineering and Technology, Future University in Egypt, New Cairo 11845, Egypt; anas.mohamed@fue.edu.eg; 10 School of Mechanical Engineering, Faculty of Engineering, Universiti Teknologi Malaysia, Johor Bahru 81310, Johor, Malaysia; asyrafriz6@gmail.com

**Keywords:** sugar palm fibre, silicone biocomposite, soft composite, mechanical properties, physical properties

## Abstract

The development of environmentally benign silicone composites from sugar palm fibre and silicone rubber was carried out in this study. The mechanical, physical, and morphological properties of the composites with sugar palm (SP) filler contents ranging from 0% to 16% by weight (wt%) were investigated. Based on the uniaxial tensile tests, it was found that the increment in filler content led to higher stiffness. Via dynamic mechanical analysis (DMA), the viscoelastic properties of the silicone biocomposite showed that the storage modulus and loss modulus increased with the increment in filler content. The physical properties also revealed that the density and moisture absorption rate increased as the filler content increased. Inversely, the swelling effect of the highest filler content (16 wt%) revealed that its swelling ratio possessed the lowest rate as compared to the lower filler addition and pure silicone rubber. The morphological analysis via scanning electron microscopy (SEM) showed that the sugar palm filler was evenly dispersed and no agglomeration could be seen. Thus, it can be concluded that the addition of sugar palm filler enhanced the stiffness property of silicone rubber. These new findings could contribute positively to the employment of natural fibres as reinforcements for greener biocomposite materials.

## 1. Introduction

The development of new materials is rapidly increasing to meet new demands in this era of advanced technologies. Research and fabrications on composite materials, especially fibre-reinforced polymer composites, are also gaining interest due to their superior strength as compared to the individual material, and they exhibit noncorrosive properties [1,2]. This type of composite material has dominated the heavy industries such as aerospace, electrical transmission systems, marine, and automotive [3,4,5,6,7,8,9]. Carbon fibres and aramid are among the famous reinforcement materials used that usually bond with matrices such as epoxy, polyester, and polypropylene [10,11,12].

These synthetic fibres, however, deal with critical issues in terms of their disposal effects due to their nonbiodegradable properties. Anthropogenic carbon dioxide is released upon burning these synthetic fibres. The gasses release leads to serious environmental issues [13,14]. Other drawbacks of synthetic fibres include nonrenewability, which may lead to health problems when inhaled, and high cost [15,16,17,18,19]. Due to the aforementioned issues, researchers are trying to overcome this by improving or replacing the existing materials with biodegradable and eco-friendly materials towards the users and the environment. Therefore, natural fibres such as kenaf, bamboo, and jute are among the chosen materials to replace the synthetic fibres in composite materials [20,21,22,23]. Natural fibres are preferred due to their renewable ability, low density, low cost, low risk to health, and abundance in nature [24,25,26].

Studies on these natural fibres have been extensively explored to investigate their physical and mechanical properties. They are normally bound with thermoplastic and thermoset polymers. To increase the use of greener materials, researchers have broadened their studies to bind the natural fibres with bio-based polymers, for example, polylactic acid and starch [27,28,29,30]. However, in this study, the authors aimed to produce a soft biocomposite material using silicone rubber as the matrix that was reinforced with natural fibre called sugar palm fibre, which is also known by its scientific name: *Arenga pinnata* fibre. The fibres are durable and possess good seawater resistance [31,32,33]. They are also cheap, abundant, and easy to collect as the fibres are warped along the tree trunk from top to bottom [34,35].

Previous studies on silicone composites have mainly been to improve their tensile strength as well as the thermal and electrical conductivity of the silicone rubber by adding fillers [36,37,38,39]. Among the chosen fillers were graphene nanoplatelets [38], nickel [40], silica [41], carbon black [42], and hollow glass microspheres [43]. Witt et al. [44] investigated the influence of carbon black and carbon nanotube fillers on silicone rubber and it was found that the tensile strength and electrical properties of the nanocomposite improved significantly. This discovery shows that the material possesses good potential for pressure sensor applications. Another study by Yang et al. [45] also reported that the addition of zirconia or zirconium carbide into silicone rubber enhanced the thermal and ablation properties. In addition to that, due to a different processing of graphite nanoplatelets, the electrical and thermal conductivities of the composite processed by the three roll-mill were considerably improved in comparison to the mechanical mixing and speed mixing techniques [46]. This shows that the dispersion state of the filler in silicone rubber is a main aspect that should be taken into consideration. From these previous studies conducted, it is shown that the addition of fillers into silicone rubber has significantly improved the mechanical, thermal, and electrical properties of the silicone rubber. These efforts have broadened the studies in the field of silicone composite materials. For that, this study found that the research on the addition of bio-filler into silicone rubber is still lacking. Mathew et al. [47] also selected a natural-based reinforcement for its rubber-based matrix, called isora fibre. It was chosen owing to its biodegradable properties, low cost, abundance in nature, and low density.

Partly to increase awareness on the environmental issues, this study chose sugar palm fibre to be added into silicone rubber for a greener composite material. The sugar palm tree has many potential uses as almost every part of the tree can be used [48]. This tree is famous for its palm sap where palm neera, *gula kabung*, and many more can be produced and commercialised [49]. Another important part of the tree is the sugar palm fibre itself as, traditionally, it has been used to make ropes, brooms, mats, and cushions. However, the demand for this product is low as it only focuses in the rural areas. Abundant sugar palm fibre residues have not been utilised, which might cause a negative effect towards nature as it is burned in the field [50]. Thus, this encouraged this study to select sugar palm fibres as a filler for silicone rubber. Many studies have also carried out investigations on the properties of the sugar palm fibres, and promising results were obtained as the properties of sugar palm fibres are also comparable to other natural fibres [51].

Therefore, this study aims to report on the mechanical properties of the sugar-palm-reinforced silicone rubber biocomposite via the uniaxial tensile test. Its viscoelastic behaviour was characterised under dynamic mechanical analysis (DMA) to determine its storage modulus, (E′), loss modulus, (E″), and tan δ, while the physical characteristics of the sugar-palm-reinforced silicone rubber biocomposite were investigated by analysing its density, moisture absorption content, and swelling behaviour. The morphology of the break specimens was also analysed using a scanning electron microscope (SEM).

## 2. Methodology

### 2.1. Material

Silicone rubber (Ecoflex 00-30 Platinum Cure) supplied by Castmech Sdn Bhd, Ipoh, Malaysia was selected as the matrix for this study. The product came with two parts: Part A and Part B. The silicone mixture was mixed using a ratio of 1:1. The sugar palm fibres were supplied by the local people in Kuala Pilah, Negeri Sembilan, Malaysia.

### 2.2. Specimen Preparation

The fibres were harvested and washed using clean water to ensure contaminants such as dry leaves and dusts were removed from the fibres. The fibres were then let to dry at room temperature for 24 h. The cleaned sugar palm fibres were then crushed using the crushing machine followed by the planetary mono mill machine (Pulverisette Classic Line 6, Fritsch, Germany). To control the size of the sugar palm (SP) filler, the final step of the fibre preparation included the sieving process using a mesh with a frame size of 0.25 mm. The sugar-palm-reinforced silicone rubber biocomposite specimens were prepared with 0 wt% (Pure silicone rubber), 4 wt%, 8 wt%, 12 wt%, and 16 wt% filler compositions. The specimens were prepared by mixing both part A and part B of the silicone rubber. After constant stirring, the weighed fillers were inserted into the mixture and the stirring process was then continued. It is crucial to achieve homogeneity by stirring the mixture evenly to ensure that no accumulated fillers are found in the mixture. The mixture was then poured into the mould and was let to cure for four hours at room temperature.

### 2.3. Mechanical Characterisation

#### 2.3.1. Uniaxial Tensile Test

The mechanical tensile behaviour of the sugar-palm-reinforced silicone rubber biocomposite was investigated using a 3382 Universal Testing Machine (Instron, Norwood, MA, USA). The test was conducted based on the ASTM D412 standard with a speed rate of 500 mm/min. For each filler composition, nine samples were tested until a failure state was reached.

#### 2.3.2. Dynamic Mechanical Analysis (DMA)

The viscoelastic behaviour of the silicone biocomposite specimens were studied via a dynamic mechanical analysis using DMA 8000 (Perkin Elmer, Waltham, MA, USA). Three specimens for each filler composition were prepared with dimensions of 30 mm × 5 mm × 3 mm. The viscoelastic behaviour of the samples was compared from the storage modulus, loss modulus, and tan delta parameters. All specimens were exposed to a tension mode with a constant frequency of 1 Hz. The test temperature was varied from room temperature up to 200 °C.

### 2.4. Physical Characterisation

#### 2.4.1. Density Test

Six specimens with dimensions of 20 mm × 20 mm × 3 mm for each composition were prepared and their density was determined using the Archimedes method. A Specific Gravity Measuring Kit (AD-1653, Cole-Parmer, Japan) was used to determine the weight of the specimens. Each specimen was weighed both in air and distilled water. Their density value was obtained from Equation (1).

(1) $$\rho =\left(\frac{A}{A-B}\right)\times \left(\rho_{O}-d\right)+d$$

where *ρ* is the density, A is the weight of the specimen in air, B is the weight of the specimen in water, *ρ_o_* is the density of distilled water (0.9973 g/cm^3^, temperature: 24 °C), and *d* is the density of air (0.001 g/cm^3^).

#### 2.4.2. Moisture Absorption Test

A similar procedure was carried out by [52] where six specimens were prepared for the moisture absorption test with dimensions of 50 mm × 50 mm × 2 mm. The specimens were then immersed in distilled water for 24 h at room temperature. The specimens were removed from distilled water, wiped, and dried thoroughly to ensure no excess of water was present on the surface. The specimen was then weighed to obtain the weight gain. The volume, % of moisture absorption was then calculated using Equation (2):

(2) $$\mathrm{Volume},\%=\frac{\left(W_{f}-W_{i}\right)/\rho_{w}}{\left(\left(W_{f}-W_{i}\right)/\rho_{w}\right)+\left(W_{i}\right)/\rho_{c}}\text{×}100$$

where *W_f_* and *W_i_* are the final and initial weights of the specimens and *ρ*_*w*_ and *ρ*_*c*_ are densities of the water and composite specimen, respectively.

#### 2.4.3. Swelling Test

Six specimens for each filler composition were prepared with dimensions of 50 mm × 25 mm × 2 mm. The test was conducted based on ASTM D471 where toluene liquid was chosen as the swelling agent. The test was carried out at room temperature. For the first 22 h of immersion, the weights of each specimen were taken for every two hours. The immersion was then continued for another 48 h with 24 h of interval. The swell rate of the specimen was then calculated using Equation (3):

(3) $$\Delta M,\;\%=\frac{M_{2}-M_{1}}{M_{1}}\text{×}100\%$$

where ΔM is the change in mass (%), *M*_1_ is the mass of the specimen before immersion (g), and *M*_2_ is the mass of the specimen after immersion (g).

### 2.5. Scanning Electron Microscope (SEM)

The morphological surface of the specimens after the tensile test was examined using a TM3000 Scanning Electron Microscope (SEM) (Hitachi, Japan). The surfaces to be examined were coated with platinum using a sputter coater machine (SC7620, Polaron, Watford, UK) to avoid the specimen from charging in order to obtain better image quality.

## 3. Results and Discussion

### 3.1. Mechanical Characterisation

#### 3.1.1. Uniaxial Tensile Test

**Figure 1:**
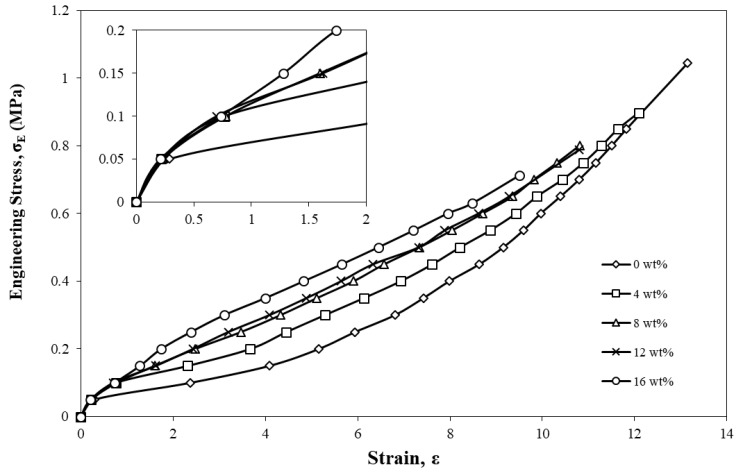
Engineering stress, σ_E_, strain, ε, of pure silicone rubber (0 wt%) and sugar-palm-reinforced silicone rubber biocomposite.

Figure 1 shows the stress–strain curve of pure silicone rubber and the sugar-palm-reinforced silicone rubber biocomposite (4 wt%, 8 wt%, 12 wt%, and 16 wt%). The curves showed the average tensile properties from nine specimens for each filler composition where the *x-* and *y*-axes of the graph represent strain, ε, and engineering stress, σ_E_, respectively. It can be observed that the curves behaved highly nonlinearly where there was a slight concave downward pattern from 0 to 0.05 MPa for all specimens, as can be seen in the inset of Figure 1. After 0.05 MPa, the pure silicone rubber and 4 wt% curves continued to increase with a concave upward pattern until it reached a failure state, while the specimens with increased filler contents of 8 wt%, 12 wt%, and 16 wt% showed that the nonlinear elastic behaviour was reduced, making it almost linear-like. It could also be observed that a further increment in the sugar palm filler into the silicone rubber showed a gradual increase in the gradient of the graph. This indicates that the stiffness of the specimen increased with the filler content. These results are consistent with Ziraki et al.’s [53] findings where the further addition of polypropylene fibres into the silicone rubber increased the specimen’s stiffness property. Gan et al. [54] reported similar outcomes where the stiffness property of the graphene nanoribbon-filled silicone rubber nanocomposite had improved significantly in comparison to pristine silicone rubber. Karthikeyan et al. also found that the addition of sisal fibre into the silicone rubber had enhanced the stiffness of the materials. This was attributed to the filler volume fraction as a higher filler content would increase the crosslink density of the silicone rubber to resist the deformation and thus increase the material’s stiffness. In addition to that, the filler distribution plays an important role in ensuring the loads are uniformly transferred [55].

Table 1 displays the average maximum tensile strength and strain values for each filler composition. From the table, pure silicone rubber possessed the highest average tensile strength of 1.04 MPa and strain of 13.15. This showed that pure silicone rubber exhibited the most flexible behaviour with the highest strain value but lacks the stiffness property as compared to the specimen with added sugar palm filler.

With the increment in SP filler content, it gradually reduced the values of maximum tensile strength and strain. Unlike synthetic fibres, natural fibres exhibit a lower strength and durability [56] and, thus, could contribute to a lower mechanical tensile strength. The load transferred from the silicone rubber to the sugar palm filler was unable to effectively be absorbed by the filler due to the lack of durability of natural fibres in withstanding the load, and this resulted in an early breakage of the specimen with lower ultimate tensile strength and strain at break. These results are consistent with those of Ismail et al. [57] who reported that the increment in bamboo fibre into natural rubber decreased the tensile strength and elongation at break of the composite. Another similar finding was reported by Berahman et al. [58] where the increment in the filler content monotonically reduced the elongation at break of the samples.

#### 3.1.2. Morphological Characterisation

**Figure 2:**
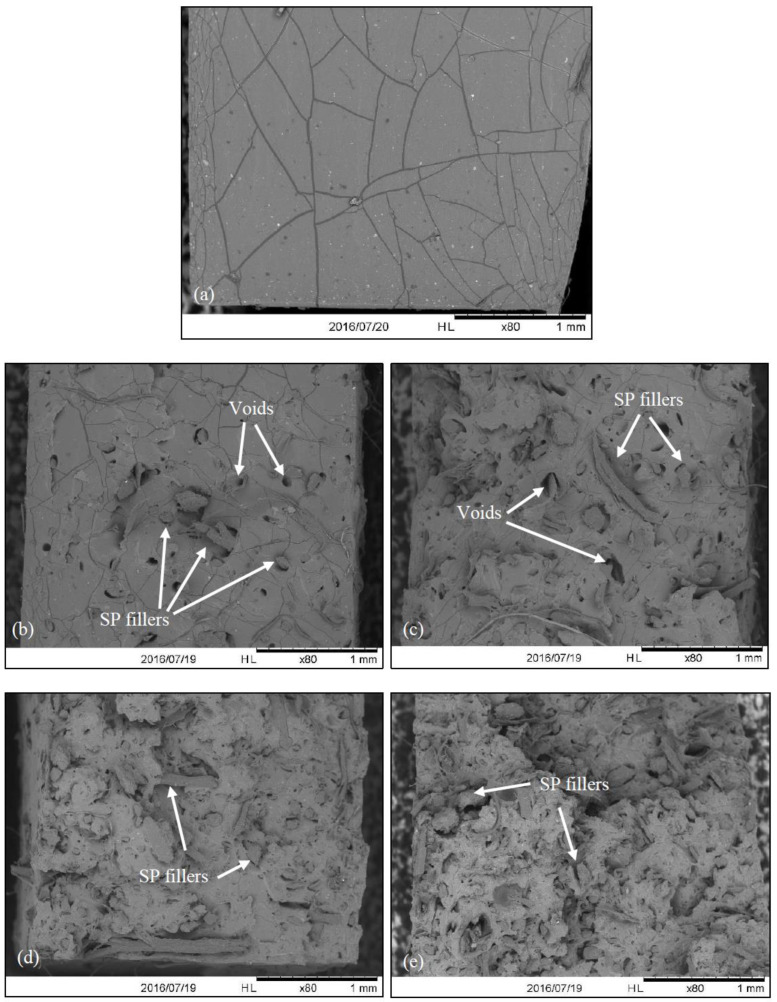
SEM images of (**a**) pure silicone rubber (0 wt%), (**b**) 4 wt%, (**c**) 8 wt%, (**d**) 12 wt%, and (**e**) 16 wt% sugar-palm-reinforced silicone rubber biocomposite.

To further support Table 1, Figure 2 shows the morphological images of the pure silicone rubber and sugar-palm-reinforced silicone rubber biocomposite after the tensile test. It could be observed that the SEM image of pure silicone rubber as shown in Figure 2a revealed a smooth surface with no formation of voids. The SEM images of specimens with added sugar palm filler (Figure 2b–e) displayed an uneven fracture surface as the surface became rougher with a higher filler loading. Despite having a low durability in SP fillers, the early breakage of the specimens could also be due to the irregular size of sugar palm fillers that caused an uneven load transfer in between the filler and the matrix. Furthermore, the addition of filler led to the formation of voids, as can be seen from the SEM images, due to the air trapped within the fillers and the matrix during the curing process. Namitha et al. [52] in their study also described a similar result. Furthermore, through the SEM images, the voids shown also proved why specimens with added sugar palm filler resulted in a significant drop in its tensile strength and strain values. However, these specimens also proved that the load was transferred effectively between the fillers and the matrix as most failure occurred at the fillers. The fillers were also well-dispersed and no SP filler agglomeration could be observed.

#### 3.1.3. Dynamic Mechanical Analysis (DMA)

**Figure 3:**
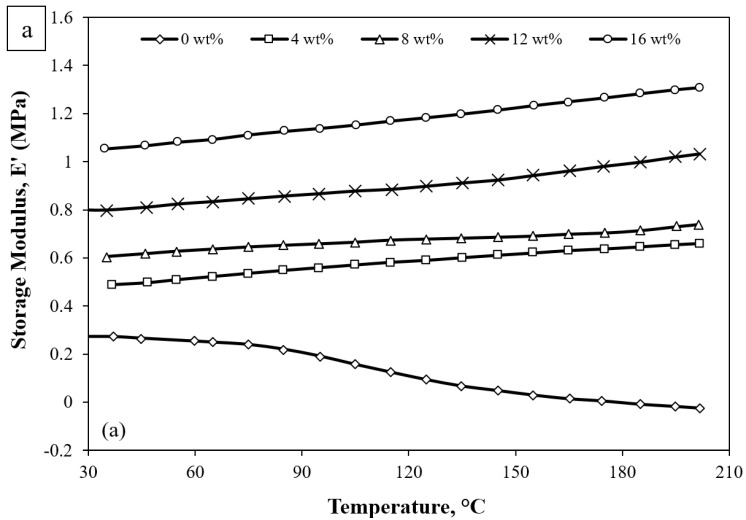

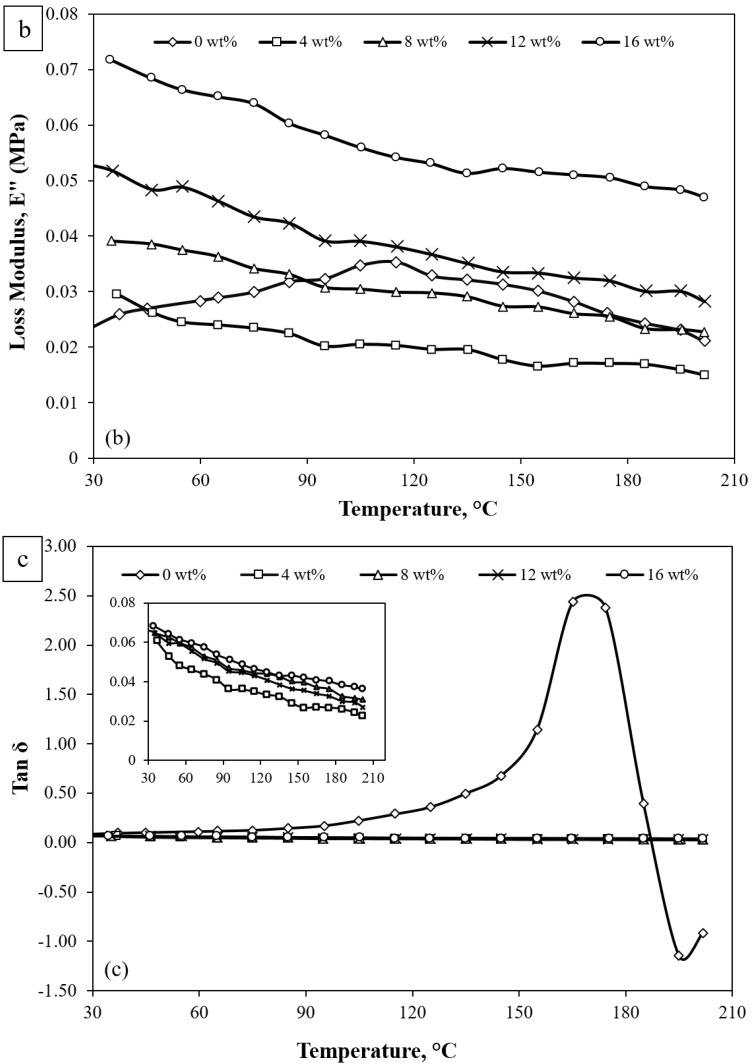
(**a**) Storage modulus, (**b**) loss modulus, and (**c**) tan delta of pure silicone rubber and sugar -palm-reinforced silicone rubber biocomposite.

The variation in the viscoelastic behaviour, storage modulus (E′), loss modulus (E″), and tan delta (tan δ) of pure silicone rubber, 4 wt%, 8 wt%, 12 wt%, and 16 wt% sugar-palm-reinforced silicone rubber biocomposites are shown in Figure 3a–c in the temperature range from room temperature to 200 °C. It could be observed that without the sugar palm filler, pure silicone rubber exhibited the lowest storage modulus property and its stiffness property decreased (reaching negative values) with an increase in operating temperature. This indicates that the specimen’s ability to retain the stiffness property by absorbing the energy received decreased across the temperature range. A similar trend could be observed where the storage modulus of specimens with added sugar palm filler increased steadily with the increase in temperature condition. It could also be seen that the stiffness property of 4 wt%, 8 wt%, 12 wt%, and 16 wt% specimens improved significantly by 70%, 100%, 190%, and 280%, respectively. These results revealed that the storage modulus was enhanced by the addition of sugar palm filler into the silicone rubber. The main cause of pure silicone rubber losing its ability to absorb the energy throughout the heating process may be due to the weak intermolecular forces among the polymer chain of -Si-O-Si- [59]. This would reduce the ability of the pure silicone rubber to store the energy upon receiving it, hence resulting in a low stiffness property. In contrast, the presence of sugar palm filler appeared to absorb most of the heat exposed by the silicone rubber and thus reduce the mobility of the silicone rubber polymer chain. This resulted in an improvement of the weak intermolecular forces of the polymer chain in the silicone rubber into stronger intermolecular forces. Thus, this improved the stiffness property of the specimen with added sugar palm filler as compared to pure silicone rubber. Furthermore, these results also showed that the interfacial adhesion between the sugar palm filler and the silicone rubber possessed good interfacial bonding as the load and heat-exposed area transferred effectively. As a result, the stiffness property was enhanced. A similar finding was also reported by Feuchtwanger et al. where the addition of NiMnGa increased the storage modulus of silicone rubber by 50% [60]. The results also showed an almost similar pattern where the storage modulus increased upon heating, especially for silicone rubber with the highest volume fraction of the bulk single crystal.

As for loss modulus, E″, Figure 3b presents the viscous part of pure silicone rubber and the sugar-palm-reinforced silicone rubber biocomposite. The pattern of the graph of all sugar-palm-reinforced silicone rubber biocomposite (4 wt%, 8 wt%, 12 wt%, and 16 wt%) specimens showed a decrease in loss modulus as the temperature increased. Interestingly, the 16 wt% specimen possessed the highest viscous portion followed by 12 wt%, 8 wt%, and 4 wt% specimens. This was due to the presence of sugar palm filler, which absorbed the energy that was transferred from the silicone rubber. This resulted in a low elastic response of silicone rubber. Therefore, this indicates that the 16 wt% specimen exhibited the highest energy lost due to friction and molecular motions [61]. Dislocation of sugar palm filler might also occur during the test, and this would be a logical explanation for the energy loss. A higher filler content would result in more energy loss as more dislocation and internal friction took place. This result is in agreement with [62] where the presence of fillers had a significant effect on the loss modulus of the composites. It could also be observed that the 8 wt% specimen exhibited lower viscous properties in comparison to the 16 wt% and 12 wt% specimens, which was almost similar tos pure silicone rubber after 164 °C. Pure silicone rubber, however, displayed a different behaviour where the loss modulus increased up to 124 °C and then continued to decrease gradually with the heating temperature. The decrease trend of silicone rubber also showed that the performance of the silicone rubber in dissipating the energy was almost similar to those of 4 wt% and 8wt% specimens after 124 °C. This might due to the lower mobility of molecular chains of the silicone rubber as the internal friction in between the filler–filler and filler–matrix is lower as compared to the 12 wt% and 16 wt% specimens [63,64,65].

The tan delta or damping ratio is the effectiveness of the material to dissipate the energy via internal friction and molecular movement. From Figure 3c, pure silicone rubber demonstrated the highest damping performance where the trend increased up to 164 °C and declined rapidly as the heating continued as compared to specimens with sugar palm filler. The pure silicone rubber showed an ineffectiveness at absorbing and dissipating the energy as it may fail abruptly where the damping ratio dropped sharply after 164 °C until it reached negative tan delta values. The specimens with added sugar palm filler, on the other hand, possessed almost similar tan delta behaviour where it decreased with the increasing temperature test, as shown in the inset of Figure 3c. However, the data revealed that the 16 wt% specimen was better at dissipating energy as compared to the 4 wt%, while the 12 wt% and 8 wt% specimens showed an almost similar damping ratio. This indicates that higher-filler-addition specimens are good at energy absorption but have less potential in dissipating the energy. Although the sugar-palm-reinforced silicone rubber biocomposite was found to exhibit low damping capacity, it still had a better damping capacity in comparison to pure silicone rubber. The effect of the addition of sugar palm filler into the silicone rubber could be seen when the tan delta behaviour was different than the pure silicone rubber as it dropped abruptly to negative tan delta values. This similar trend of sugar-palm-reinforced silicone rubber biocomposites has also been reported in the previous literature [60,66,67] where the damping ratio decreases as the heating continues. The interactions of filler–matrix and filler–filler play an important role in storing the energy and dissipating it effectively. From the data obtained, it can be concluded that the addition of sugar palm filler decreases the mobility of the polymer chain of the silicone rubber and it shows that the sugar palm filler and silicone rubber have a strong adhesion as it tends to store the energy rather than dissipate it. This theory is in good agreement with Kamaruddin et al. [64] as the graphene content increases into the rubber band.

### 3.2. Physical Properties

#### 3.2.1. Density Measurement

**Figure 4:**
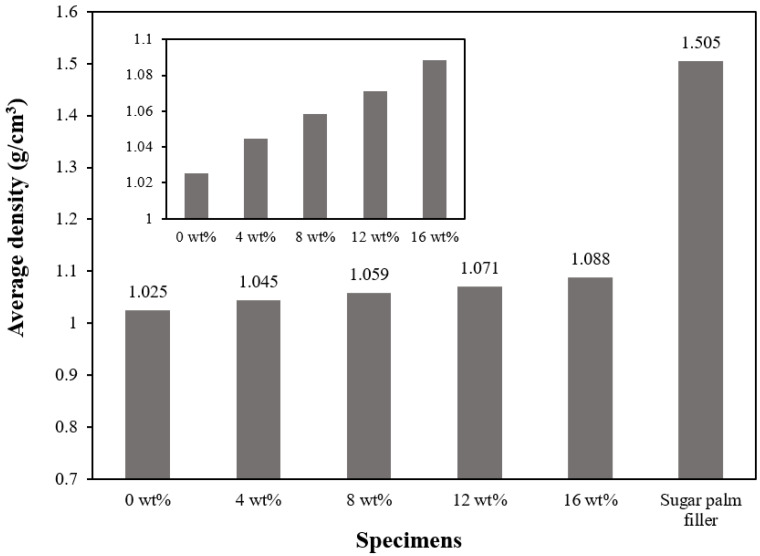
Experimental density of pure silicone rubber, sugar palm filler, and sugar-palm-reinforced silicone rubber biocomposite.

Figure 4 illustrates the experimental density of the composites with various filler contents compared to the density of sugar palm filler. Compared to pure silicone rubber with an experimental density of 1.025 g/cm^3^, the sugar-palm-reinforced silicone rubber biocomposite’s experimental density increased linearly with the filler content, as depicted in the inset of Figure 4. The increase in density was similar to the findings of Gao et al. [68] where the increment in filler content increased the density of the silicone rubber foam. Furthermore, Sanyang et al. [69] also found that the density of sugar palm starch/polylactic acid bilayer films increased significantly to 1.35 g/cm^3^ with loadings up to 80 wt%. Another similar trend could also be observed in a study by Atikah et al. [70]. It was reported that when glass fibre was added into the sugar-palm-fibre-reinforced epoxy composite, the composite’s density increased up to 1.4 g/cm^3^. In comparison, it revealed that the highest density value (16 wt%) in this study was actually much lower as compared to the saturated specimens of Sanyang’s and Atikah’s findings.

#### 3.2.2. Moisture Absorption Behaviour

**Figure 5:**
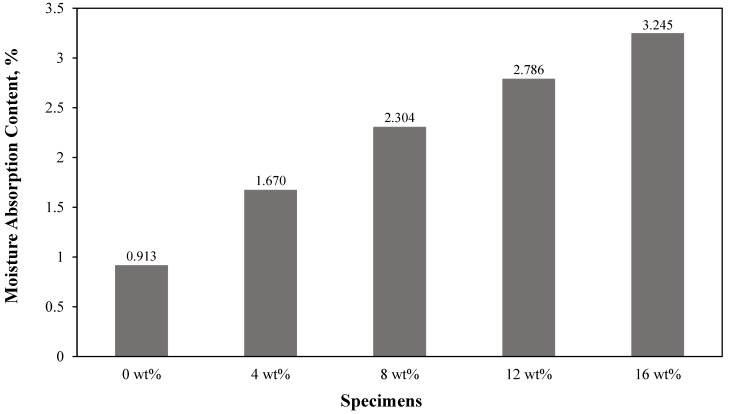
Moisture absorption of pure silicone rubber and sugar-palm-reinforced silicone rubber biocomposite.

Figure 5 presents the average moisture absorption content for each specimen after 24 h of immersion in distilled water. Clearly, the results showed an increase in moisture absorption with the increment in filler content. The 16 wt% specimens exhibited the highest moisture absorption content of up to 3.245%, while pure silicone rubber possessed the lowest. Similar results were reported by Namitha et al. [71] where a higher volume of ceramic in silicone rubber led to a higher water intake. Obviously in this study, natural fibres are known for their hydrophilic properties as they are mainly composed of cellulose and hemicellulose layers. This natural hydrophilic behaviour that exists in natural fibres allows the specimens to absorb more moisture. Thus, a higher water absorption of the specimen was obtained as a higher filler content was further added. A similar increment in moisture absorption content on other matrices using sugar palm fibre as the reinforcement or filler was also reported. Atikah et al. [70] found that the incorporation of sugar palm fibre increased the moisture intake of the hybrid composite of up to 9%. In contrast, Sahari et al. [50] claimed that the increment in AP fibre (0–30 wt%) into plasticised sugar palm starch gradually reduced the moisture content from 25% to 14%. From this study, the highest filler addition resulted in a moisture content of 3.245%, which is very much lower than Sahari’s findings. This is due to the hydrophobic property that is present in the silicone rubber that has the ability to repel water [71].

#### 3.2.3. Swelling

**Figure 6:**
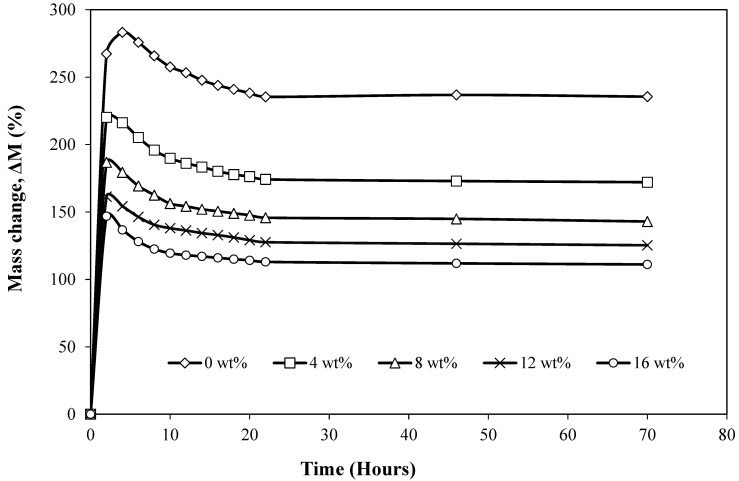
Swelling behaviour of both pure silicone rubber and sugar-palm-reinforced silicone rubber biocomposite with 4 wt%, 8 wt%, 12 wt%, and 16 wt% of filler content.

Figure 6 displays the average swelling behaviour of both pure silicone rubber and the sugar-palm-reinforced silicone rubber biocomposite with 4 wt%, 8 wt%, 12 wt%, and 16 wt% of filler content. The results displayed the same pattern where for the first two hours, the swelling rate increased significantly. This indicates the specimens’ ability to fully absorb the toluene liquid. Unlike the sugar-palm-reinforced silicone rubber biocomposite, pure silicone rubber continued to absorb more toluene for the next two hours, which made the curve rise with a new peak. Interestingly, it was found that the addition of 4 wt% of filler content significantly decreased the swelling ratio of the specimen as compared to the pure silicone rubber. This trend continued to decrease with the further increment in filler content where the highest filler content (16 wt%) showed the lowest swelling rate. The pattern of swell rate also showed that all specimens reached an equilibrium state after 22 h of immersion.

The highest swelling ratio from the pure silicone rubber is due to their weak intermolecular forces, and this increases the ability of the chain mobility in silicone rubber [72]. Moreover, single bonds in the Si-O-Si backbone chain of the silicone rubber will allow for more spaces to be available, thus resulting in the highest rate of swelling. In contrast, the addition of sugar palm filler certainly decreased the spaces for chain mobility to occur in silicone rubber as sugar palm dominated the spaces. This causes the movement of the chains to be restricted. As a result, this reduces the swelling rate of the sugar-palm-reinforced silicone rubber biocomposite specimens. This chain motion restriction is a result from the increase in crosslink density in silicone rubber [73,74]. This indicates that pure silicone rubber possessed the lowest crosslink density in comparison to the added sugar palm filler into the silicone rubber. Similar findings were also reported by Mostafa et al. [75] where the increment of carbon black content decreased the swelling rate induced by the higher crosslink density [75].

## 4. Conclusions

This study aimed to promote a bio-friendly composite material using sugar palm fibre as the filler and silicone rubber as the binder. From this study, the mechanical, physical, and morphological analysis of sugar-palm-reinforced silicone rubber biocomposite were analysed and it was found that the addition of sugar palm filler into silicone rubber increased the stiffness, density, as well as the moisture absorption content of the material significantly. The swelling test revealed that the increment in filler composition showed a lower swelling rate as compared to pure silicone rubber. The morphological images also displayed a good filler–matrix bonding where no agglomeration was observed. In conclusion, this study proves that the newly developed biomaterial, the sugar-palm-reinforced silicone rubber biocomposite, has great potential for many applications. Therefore, this study has enhanced significant knowledge pertaining to silicone biocomposite materials.

## Figures and Tables

**Table 1:** Average maximum tensile strength, σ_E_ (MPa), and strain, ε, of pure silicone rubber (0 wt%) and sugar-palm-reinforced silicone rubber biocomposite.

Specimens	Average Maximum Tensile Strength, σ_E_ (MPa)	Average Maximum Strain, ε
0 wt%	1.04 ± 0.05	13.15 ± 0.33
4 wt%	0.89 ± 0.02	12.09 ± 0.13
8 wt%	0.80 ± 0.02	10.81 ± 0.16
12 wt%	0.79 ± 0.03	10.79 ± 0.22
16 wt%	0.71 ± 0.02	9.49 ± 0.17

## Data Availability

Not applicable.

## References

[1] Cheung H., Ho M., Lau K., Cardona F., Hui D. (2009). Natural fibre-reinforced composites for bioengineering and environmental engineering applications. Compos. Part B Eng..

[2] Ilyas R.A., Zuhri M.Y.M., Norrrahim M.N.F., Misenan M.S.M., Jenol M.A., Samsudin S.A., Nurazzi N.M., Asyraf M.R.M., Supian A.B.M., Bangar S.P. (2022). Natural Fiber-Reinforced Polycaprolactone Green and Hybrid Biocomposites for Various Advanced Applications. Polymers.

[3] Borrego L.P., Costa J.D.M., Ferreira J.A.M., Silva H. (2014). Fatigue behaviour of glass fibre reinforced epoxy composites enhanced with nanoparticles. Compos. Part B Eng..

[4] Singh H., Namala K.K., Mahajan P. (2015). A damage evolution study of E-glass/epoxy composite under low velocity impact. Compos. Part B Eng..

[5] Asyraf M.R.M., Ishak M.R., Sapuan S.M., Yidris N., Rafidah M., Ilyas R.A., Razman M.R. (2020). Potential application of green composites for cross arm component in transmission tower: A brief review. Int. J. Polym. Sci..

[6] Asyraf M.R.M., Ishak M.R., Syamsir A., Amir A.L., Nurazzi N.M., Norrrahim M.N.F., Asrofi M., Rafidah M., Ilyas R.A., Rashid M.Z.A. (2022). Filament-wound glass-fibre reinforced polymer composites: Potential applications for cross arm structure in transmission towers. Polym. Bull..

[7] Asyraf M.R.M., Ishak M.R., Norrrahim M.N.F., Amir A.L., Nurazzi N.M., Ilyas R.A., Asrofi M., Rafidah M., Razman M.R. (2022). Potential of Flax Fibre Reinforced Biopolymer Composites for Cross-Arm Application in Transmission Tower: A Review. Fibers Polym..

[8] Asyraf M.R.M., Ishak M.R., Sapuan S.M., Yidris N., Ilyas R.A. (2020). Woods and composites cantilever beam: A comprehensive review of experimental and numerical creep methodologies. J. Mater. Res. Technol..

[9] Asyraf M.R.M., Syamsir A., Zahari N.M., Supian A.B.M., Ishak M.R., Sapuan S.M., Sharma S., Rashedi A., Razman M.R., Zakaria S.Z.S. (2022). Product Development of Natural Fibre-Composites for Various Applications: Design for Sustainability. Polymers.

[10] Asyraf M.R.M., Ishak M.R., Sapuan S.M., Yidris N. (2021). Utilization of Bracing Arms as Additional Reinforcement in Pultruded Glass Fiber-Reinforced Polymer Composite Cross-Arms: Creep Experimental and Numerical Analyses. Polymers.

[11] Asyraf M.R.M., Ishak M.R., Sapuan S.M., Yidris N. (2021). Comparison of Static and Long-term Creep Behaviors between Balau Wood and Glass Fiber Reinforced Polymer Composite for Cross-arm Application. Fibers Polym..

[12] Alhayek A., Syamsir A., Supian A.B.M., Usman F., Asyraf M.R.M., Atiqah M.A. (2022). Flexural Creep Behaviour of Pultruded GFRP Composites Cross-Arm: A Comparative Study on the Effects of Stacking Sequence. Polymers.

[13] Roslan Z.B., Ramli Z., Razman M.R., Asyraf M.R.M., Ishak M.R., Ilyas R.A., Nurazzi N.M. (2021). Reflections on Local Community Identity by Evaluating Heritage Sustainability Protection in Jugra, Selangor, Malaysia. Sustainability.

[14] Ali S.S.S., Razman M.R., Awang A., Asyraf M.R.M., Ishak M.R., Ilyas R.A., Lawrence R.J. (2021). Critical Determinants of Household Electricity Consumption in a Rapidly Growing City. Sustainability.

[15] Sanyang M.L., Sapuan S.M., Jawaid M., Ishak M.R., Sahari J. (2016). Recent developments in sugar palm (Arenga pinnata) based biocomposites and their potential industrial applications: A review. Renew. Sustain. Energy Rev..

[16] Ilyas R.A., Aisyah H.A., Nordin A.H., Ngadi N., Zuhri M.Y.M., Asyraf M.R.M., Sapuan S.M., Zainudin E.S., Sharma S., Abral H. (2022). Natural-Fiber-Reinforced Chitosan, Chitosan Blends and Their Nanocomposites for Various Advanced Applications. Polymers.

[17] Nurazzi N.M., Sabaruddin F.A., Harussani M.M., Kamarudin S.H., Rayung M., Asyraf M.R.M., Aisyah H.A., Norrrahim M.N.F., Ilyas R.A., Abdullah N. (2021). Mechanical Performance and Applications of CNTs Reinforced Polymer Composites—A Review. Nanomaterials.

[18] Nurazzi N.M., Asyraf M.R.M., Fatimah Athiyah S., Shazleen S.S., Rafiqah S.A., Harussani M.M., Kamarudin S.H., Razman M.R., Rahmah M., Zainudin E.S. (2021). A Review on Mechanical Performance of Hybrid Natural Fiber Polymer Composites for Structural Applications. Polymers.

[19] Mohd Nurazzi N.M., Muhammad Asyraf M.R.M., Khalina A., Abdullah N., Sabaruddin F.A., Kamarudin S.H., Ahmad S., Mahat A.M., Lee C.L., Aisyah H.A. (2021). Fabrication, Functionalization, and Application of Carbon Nanotube-Reinforced Polymer Composite: An Overview. Polymers.

[20] Asyraf M.R.M., Ishak M.R., Syamsir A., Nurazzi N.M., Sabaruddin F.A., Shazleen S.S., Norrrahim M.N.F., Rafidah M., Ilyas R.A., Rashid M.Z.A. (2022). Mechanical properties of oil palm fibre-reinforced polymer composites: A review. J. Mater. Res. Technol..

[21] Asyraf M.R.M., Ishak M.R., Norrrahim M.N.F., Nurazzi N.M., Shazleen S.S., Ilyas R.A., Rafidah M., Razman M.R. (2021). Recent advances of thermal properties of sugar palm lignocellulosic fibre reinforced polymer composites. Int. J. Biol. Macromol..

[22] Asyraf M.R.M., Rafidah M., Azrina A., Razman M.R. (2021). Dynamic mechanical behaviour of kenaf cellulosic fibre biocomposites: A comprehensive review on chemical treatments. Cellulose.

[23] Norizan M.N., Norrrahim M.N.F., Sabaruddin F.A., Rushdan A.I., Hua L.S., Mohammad Padzil F.N., Abdul Ghani M.A., Shazleen S.S., Alias A.H., Mohidem N.A. (2022). Mechanical Performance Evaluation of Bamboo Fibre Reinforced Polymer Composites and its Applications: A Review. Funct. Compos. Struct..

[24] Kamarul Bahrain S.H., Mahmud J., Rasid Z.A., Madete J. (2017). Synthesization and Microstructural Analysis of Arenga Pinnata Fibres and Silicone Rubber for New Silicone Biocomposite Material. J. Mech. Eng..

[25] Selvan M.T.G.A., Binoj J.S., Moses J.T.E.J., Sai N.P., Siengchin S., Sanjay M.R., Liu Y. (2022). Extraction and characterization of natural cellulosic fiber from fragrant screw pine prop roots as potential reinforcement for polymer composites. Polym. Compos..

[26] Ilyas R.A., Sapuan S.M., Atiqah A., Ibrahim R., Abral H., Ishak M.R., Zainudin E.S., Nurazzi N.M., Atikah M.S.N., Ansari M.N.M. (2020). Sugar palm (*Arenga pinnata* [Wurmb.] Merr) starch films containing sugar palm nanofibrillated cellulose as reinforcement: Water barrier properties. Polym. Compos..

[27] Ilyas R.A., Sapuan S.M., Harussani M.M., Hakimi M.Y.A.Y., Haziq M.Z.M., Atikah M.S.N., Asyraf M.R.M., Ishak M.R., Razman M.R., Nurazzi N.M. (2021). Polylactic Acid (PLA) Biocomposite: Processing, Additive Manufacturing and Advanced Applications. Polymers.

[28] Duc F., Bourban P.E., Plummer C.J.G., Månson J.A.E. (2014). Damping of thermoset and thermoplastic flax fibre composites. Compos. Part A Appl. Sci. Manuf..

[29] Sahari J., Sapuan S.M., Zainudin E.S., Maleque M.A. (2012). A New Approach to Use Arenga Pinnata as Sustainable Biopolymer: Effects of Plasticizers on Physical Properties. Procedia Chem..

[30] Ilyas R.A., Zuhri M.Y.M., Aisyah H.A., Asyraf M.R.M., Hassan S.A., Zainudin E.S., Sapuan S.M., Sharma S., Bangar S.P., Jumaidin R. (2022). Natural Fiber-Reinforced Polylactic Acid, Polylactic Acid Blends and Their Composites for Advanced Applications. Polymers.

[31] Kamarul Bahrain S.H., Mahmud J., Ismail M.H. (2018). Arenga Pinnata—Silicone Biocomposite Properties via Experimental and Numerical Analysis. Mater. Sci..

[32] Nurazzi N.M., Asyraf M.R.M., Rayung M., Norrrahim M.N.F., Shazleen S.S., Rani M.S.A., Shafi A.R., Aisyah H.A., Radzi M.H.M., Sabaruddin F.A. (2021). Thermogravimetric Analysis Properties of Cellulosic Natural Fiber Polymer Composites: A Review on Influence of Chemical Treatments. Polymers.

[33] Ilyas R., Sapuan S., Atikah M., Asyraf M., Rafiqah S.A., Aisyah H., Nurazzi N.M., Norrrahim M. (2021). Effect of hydrolysis time on the morphological, physical, chemical, and thermal behavior of sugar palm nanocrystalline cellulose (Arenga pinnata (Wurmb.) Merr). Text. Res. J..

[34] Rashid B., Leman Z., Jawaid M., Ghazali M.J., Ishak M.R. (2016). Physicochemical and thermal properties of lignocellulosic fiber from sugar palm fibers: Effect of treatment. Cellulose.

[35] Nurazzi N.M., Khalina A., Chandrasekar M., Aisyah H.A., Rafiqah S.A., Ilyas R.A., Hanafee Z.M. (2020). Effect of fiber orientation and fiber loading on the mechanical and thermal properties of sugar palm yarn fiber reinforced unsaturated polyester resin composites. Polimery.

[36] Yan F., Zhang X., Liu F., Li X., Zhang Z. (2015). Adjusting the properties of silicone rubber filled with nanosilica by changing the surface organic groups of nanosilica. Compos. Part B Eng..

[37] Raza M.A., Westwood A., Brown A., Hondow N., Stirling C. (2011). Characterisation of graphite nanoplatelets and the physical properties of graphite nanoplatelet/silicone composites for thermal interface applications. Carbon.

[38] Song Y., Yu J., Yu L., Alam F.E., Dai W., Li C., Jiang N. (2015). Enhancing the thermal, electrical, and mechanical properties of silicone rubber by addition of graphene nanoplatelets. Mater. Des..

[39] Bae J.H., Chang S.H. (2013). A study on the mechanical behavior of silicone-organically modified montmorillonite composite under human body simulated environment. Compos. Sci. Technol..

[40] Boudenne A., Mamunya Y., Levchenko V., Garnier B., Lebedev E. (2015). Improvement of thermal and electrical properties of Silicone-Ni composites using magnetic field. Eur. Polym. J..

[41] Kulik V.M., Boiko A.V., Bardakhanov S.P., Park H., Chun H.H., Lee I. (2011). Viscoelastic properties of silicone rubber with admixture of SiO2 nanoparticles. Mater. Sci. Eng. A.

[42] Yi W., Wang Y., Wang G., Tao X. (2012). Investigation of carbon black/silicone elastomer/dimethylsilicone oil composites for flexible strain sensors. Polym. Test..

[43] Hu Y., Mei R., An Z., Zhang J. (2013). Silicon rubber/hollow glass microsphere composites: Influence of broken hollow glass microsphere on mechanical and thermal insulation property. Compos. Sci. Technol..

[44] Witt N., Tang Y., Ye L., Fang L. (2013). Silicone rubber nanocomposites containing a small amount of hybrid fillers with enhanced electrical sensitivity. Mater. Des..

[45] Yang D., Zhang W., Jiang B., Guo Y. (2013). Silicone rubber ablative composites improved with zirconium carbide or zirconia. Compos. Part A Appl. Sci. Manuf..

[46] Raza M.A., Westwood A.V.K., Brown A.P., Stirling C. (2012). Texture, transport and mechanical properties of graphite nanoplatelet/silicone composites produced by three roll mill. Compos. Sci. Technol..

[47] Mathew L., Joseph K.U., Joseph R. (2006). Swelling behaviour of isora/natural rubber composites in oils used in automobiles. Bull. Mater. Sci..

[48] Ishak M.R., Sapuan S.M., Leman Z., Rahman M.Z.A., Anwar U.M.K., Siregar J.P. (2013). Sugar palm (Arenga pinnata): Its fibres, polymers and composites. Carbohydr. Polym..

[49] Mukhtar I., Leman Z., Ishak M.R., Zainudin E.S. (2016). Sugar palm fibre and its composites: A review of recent developments. BioResources.

[50] Sahari J., Sapuan S.M., Zainudin E.S., Maleque M.A. (2013). Mechanical and thermal properties of environmentally friendly composites derived from sugar palm tree. Mater. Des..

[51] Norizan M.N., Abdan K., Salit M.S., Mohamed R. (2017). Physical, mechanical and thermal properties of sugar palm yarn fibre loading on reinforced unsaturated polyester composites. J. Phys. Sci..

[52] Namitha L.K., Chameswary J., Ananthakumar S., Sebastian M.T. (2013). Effect of micro- and nano-fillers on the properties of silicone rubber-alumina flexible microwave substrate. Ceram. Int..

[53] Ziraki S., Zebarjad S.M., Hadianfard M.J. (2016). A study on the tensile properties of silicone rubber/polypropylene fibers/silica hybrid nanocomposites. J. Mech. Behav. Biomed. Mater..

[54] Gan L., Shang S., Yuen C.W.M., Jiang S., Luo N.M. (2015). Facile preparation of graphene nanoribbon filled silicone rubber nanocomposite with improved thermal and mechanical properties. Compos. Part B Eng..

[55] Karthikeyan R., Tjong J., Nayak S.K., Sain M.M. (2017). Mechanical properties and cross-linking density of short sisal fiber reinforced silicone composites. BioResources.

[56] Pickering K.L., Efendy M.G.A., Le T.M. (2016). A review of recent developments in natural fibre composites and their mechanical performance. Compos. Part A Appl. Sci. Manuf..

[57] Ismail H., Edyham M.R., Wirjosentono B. (2002). Bamboo fibre filled natural rubber composites: The effects of filler loading and bonding agent. Polym. Test..

[58] Berahman R., Raiati M., Mehrabi Mazidi M., Paran S.M.R. (2016). Preparation and characterization of vulcanized silicone rubber/halloysite nanotube nanocomposites: Effect of matrix hardness and HNT content. Mater. Des..

[59] Xu Q., Pang M., Zhu L., Zhang Y., Feng S. (2010). Mechanical properties of silicone rubber composed of diverse vinyl content silicone gums blending. Mater. Des..

[60] Feuchtwanger J., Seif E., Sratongon P., Hosoda H., Chernenko V.A. (2018). Vibration damping of Ni-Mn-Ga/silicone composites. Scr. Mater..

[61] Jawaid M., Abdul Khalil H.P.S., Alattas O.S. (2012). Woven hybrid biocomposites: Dynamic mechanical and thermal properties. Compos. Part A Appl. Sci. Manuf..

[62] Singh S., Pal K. (2015). Effect of surface modified silicon carbide particles with Al_2_O_3_ and nanocrystalline spinel ZnAl_2_O_4_ on mechanical and damping properties of the composite. Mater. Sci. Eng. A.

[63] Zhou H., Song L., Lu A., Jiang T., Yu F., Wang X. (2016). Influence of immobilized rubber on the non-linear viscoelasticity of filled silicone rubber with different interfacial interaction of silica. RSC Adv..

[64] Kamaruddin N.H. (2019). Synergistic effects of rubber band infused graphene nanocomposite on morphology, spectral, and dynamic mechanical properties. Polym. Compos..

[65] Wang X., Wu L., Yu H., Xiao T., Li H., Yang J. (2020). Modified silica-based isoprene rubber composite by a multi-functional silane: Preparation and its mechanical and dynamic mechanical properties. Polym. Test..

[66] Kulshrestha U., Gupta T., Kumawat P., Jaiswal H., Ghosh S.B., Sharma N.N. (2020). Cellulose nanofibre enabled natural rubber composites: Microstructure, curing behaviour and dynamic mechanical properties. Polym. Test..

[67] Lee S., Sohn Y., Chu K., Kim D., Park S., Bae M., Kim D., Kim Y., Han I., Kim H. (2016). Suppression of negative temperature coefficient of resistance of multiwalled nanotube/silicone rubber composite through segregated conductive network and its application to laser-printing fusing element. Org. Electron..

[68] Gao J., Wang J., Xu H., Wu C. (2013). Preparation and properties of hollow glass bead filled silicone rubber foams with low thermal conductivity. Mater. Des..

[69] Sanyang M.L., Sapuan S.M., Jawaid M., Ishak M.R., Sahari J. (2016). Development and characterization of sugar palm starch and poly(lactic acid) bilayer films. Carbohydr. Polym..

[70] Afzaluddin A., Jawaid M., Salit M.S., Ishak M.R. (2019). Physical and mechanical properties of sugar palm/glass fiber reinforced thermoplastic polyurethane hybrid composites. J. Mater. Res. Technol..

[71] Namitha L.K., Sebastian M.T. (2017). High permittivity ceramics loaded silicone elastomer composites for flexible electronics applications. Ceram. Int..

[72] Zhang H., Cloud A. The permeability characteristics of silicone rubber. Proceedings of the Global Advances in Materials and Process Engineering.

[73] Feng L., Li S., Feng S. (2017). Preparation and characterization of silicone rubber with high modulus via tension spring-type crosslinking. RSC Adv..

[74] Bahrain S.H.K., Mahmud J. (2019). Swelling behaviour and morphological analysis of Arenga pinnata–silicone biocomposite. Mater. Lett..

[75] Mostafa A., Abouel-Kasem A., Bayoumi M.R., El-Sebaie M.G. (2009). Effect of carbon black loading on the swelling and compression set behavior of SBR and NBR rubber compounds. Mater. Des..

